# Testing the Impact of Prey Presentation Method on the Feeding Kinematics of Terrestrial and Aquatic Ambystomatidae

**DOI:** 10.1002/jez.70028

**Published:** 2025-08-20

**Authors:** Isabelle Toussaint‐Lardé, Vivien Louppe, Morgane Fournier, Julien Clavel, Anthony Herrel, Anne‐Claire Fabre

**Affiliations:** ^1^ Institute of Ecology and Evolution Universität Bern Bern Switzerland; ^2^ Naturhistorisches Museum Bern Bern Switzerland; ^3^ Mécanismes Adaptatifs et Evolution, UMR 7179, Muséum national d'Histoire naturelle CNRS Paris France; ^4^ Laboratoire d'Ecologie des Hydrosystèmes Naturels et Anthropisés Université Lyon 1, CNRS, UMR 5023 ‐ LEHNA Villeurbanne France; ^5^ Department of Biology, Evolutionary Morphology of Vertebrates Ghent University Ghent Belgium; ^6^ Department of Biology University of Antwerp Wilrijk Belgium; ^7^ Department of Life Sciences Natural History Museum London UK

**Keywords:** feedback control, feedforward control, jaw prehension, prey position, salamander, suction feeding, tongue prehension

## Abstract

In animal behavior, standardizing experimental protocols ensures a rigorous interpretation of the results. However, when working with multisource data, homogeneous standardization is often difficult to attain. This is the case for many studies on feeding kinematics where experimental protocols are carried out using similar but not identical methods. In particular, the impact of the way in which prey is presented remains poorly tested and understood. The aim of this study is to assess whether prey position and the way prey is presented have an impact on the kinematics of prey capture. To do so, we compared the feeding kinematics during the capture of prey presented on the substrate versus prey suspended from tweezers using closely related species of aquatic and terrestrial salamanders. Our results show that changes in prey presentation method directly impact suction feeding kinematics but not terrestrial feeding. In the case of suction feeding, when the prey is suspended by tweezers, mouth opening movements are wider and take more time, the maximum speed and acceleration of mouth opening are higher, and head angle is larger. These changes in kinematics are interpreted as behavioral responses to hydrodynamic changes caused by the different prey presentation methods. This differential sensitivity to prey presentation method between aquatic and terrestrial feeding also highlights differences in the underlying control mechanism: while terrestrial feeding appears to rely on feedback mechanisms, aquatic feeding appears to rely mostly on feedforward mechanisms. As a result, the importance of accounting for prey presentation method is likely context‐dependent, being more relevant when studying feeding systems that rely on feedforward control. Finally, when comparing aquatic and terrestrial feeding, the differences in feeding strategies due to the medium itself outweigh the effects of the prey presentation method.

## Summary

- Prey presentation method impacts suction feeding but not terrestrial feeding.
- Suction feeding relies on feedforward control and as a consequence is sensitive to changes in prey presentation method that modify the hydrodynamics of prey capture.
- Feeding on land relies mostly on feedback control and is less constrained which may explain the lack of differences in kinematics due to the prey presentation method.

## Résumé

Dans le domaine du comportement animal, la standardisation des protocoles expérimentaux garantit une interprétation rigoureuse des résultats. Cependant, lorsqu'on travaille avec des données de sources multiples, il est souvent difficile d'obtenir une standardisation homogène. C'est le cas de nombreuses études sur la cinématique de la prise alimentaire où les protocoles expérimentaux sont réalisés avec des méthodes similaires mais non identiques. En particulier, l'impact de la manière dont la proie est présentée reste mal testé et mal compris. L'objectif de cette étude est d'évaluer si la position de la proie et la façon dont elle est présentée ont un impact sur la cinématique de la capture de la proie. Pour ce faire, nous avons comparé la cinématique de la capture d'une proie présentée sur le substrat et celle de la capture d'une proie suspendue par une pince en utilisant des espèces phylogénétiquement proches de salamandres aquatiques et terrestres. Nos résultats montrent que les changements dans les conditions de présentation des proies ont un impact direct sur la cinématique de la capture de proie par succion, mais pas sur la cinématique de la capture terrestre de proie. Dans le cas de capture de proie par succion, lorsque la proie est suspendue par des pinces, les mouvements d'ouverture de la bouche sont plus amples et prennent plus de temps, la vitesse maximale et l'accélération de l'ouverture de la bouche sont plus élevées, et l'angle de la tête est plus important. Ces changements dans la cinématique sont interprétés comme des réponses comportementales aux changements hydrodynamiques causés par les différentes conditions d'offre de la proie.

Cette différence de sensibilité à la méthode de présentation des proies entre l'alimentation aquatique et terrestre met également en évidence des différences dans les mécanismes de contrôle sous‐jacents: tandis que la prise alimentaire terrestre semble reposer sur des mécanismes de rétrocontrôle (feedback), la prise alimentaire aquatique semble s'appuyer principalement sur des mécanismes de commande anticipée (feedforward). Par conséquent, l'importance de prendre en compte la méthode de présentation des proies dépend probablement du contexte, elle est plus pertinente dans l'étude de prises alimentaires reposant sur un contrôle feedforward. Mais, lorsqu'on compare prises alimentaires aquatique et terrestre, les différences de stratégies alimentaires liées au milieu dépassent les effets liés à la méthode de présentation des proies.

## 1 Introduction

The acquisition of in vivo data on animal behavior is of primary importance in the field of evolutionary biology, as it can provide a better understanding of the interplay between genetics, morphology, function, behavior, and external factors that impact the fitness of organisms. However, gathering such data often requires to work under different conditions in the field or in laboratory, with species with different personalities and behaviors, as well as with different diet or habitat use. In animal behavior studies, standardizing the experimental protocol ensure a rigorous interpretation of the observations (Moyaho and Beristain‐Castillo 2019). As such, unwanted factors have to be removed, or controlled, to be sure they do not impact the behavioral response of the studied organisms (Moyaho and Beristain‐Castillo 2019). In this regard, working with multisource data i.e., data collected by different operators and/or in different conditions‐ is challenging. Indeed, it increases the risk of introducing new factors that may affect the behavior of interest. The same issues appear when comparing the results of different authors who use similar, but not identical, protocols. So far, only a handful of studies have explored the impact of experimental protocol on animal behavior (Moyaho and Beristain‐Castillo 2019; Arroyo‐Araujo et al. 2022; Richter et al. 2011), yet with the development of the field of macroevolution often comparing dozens of ecologically and morphologically different species, such studies could help identify potential pitfalls and refine methodological approaches.

**Figure 1:**
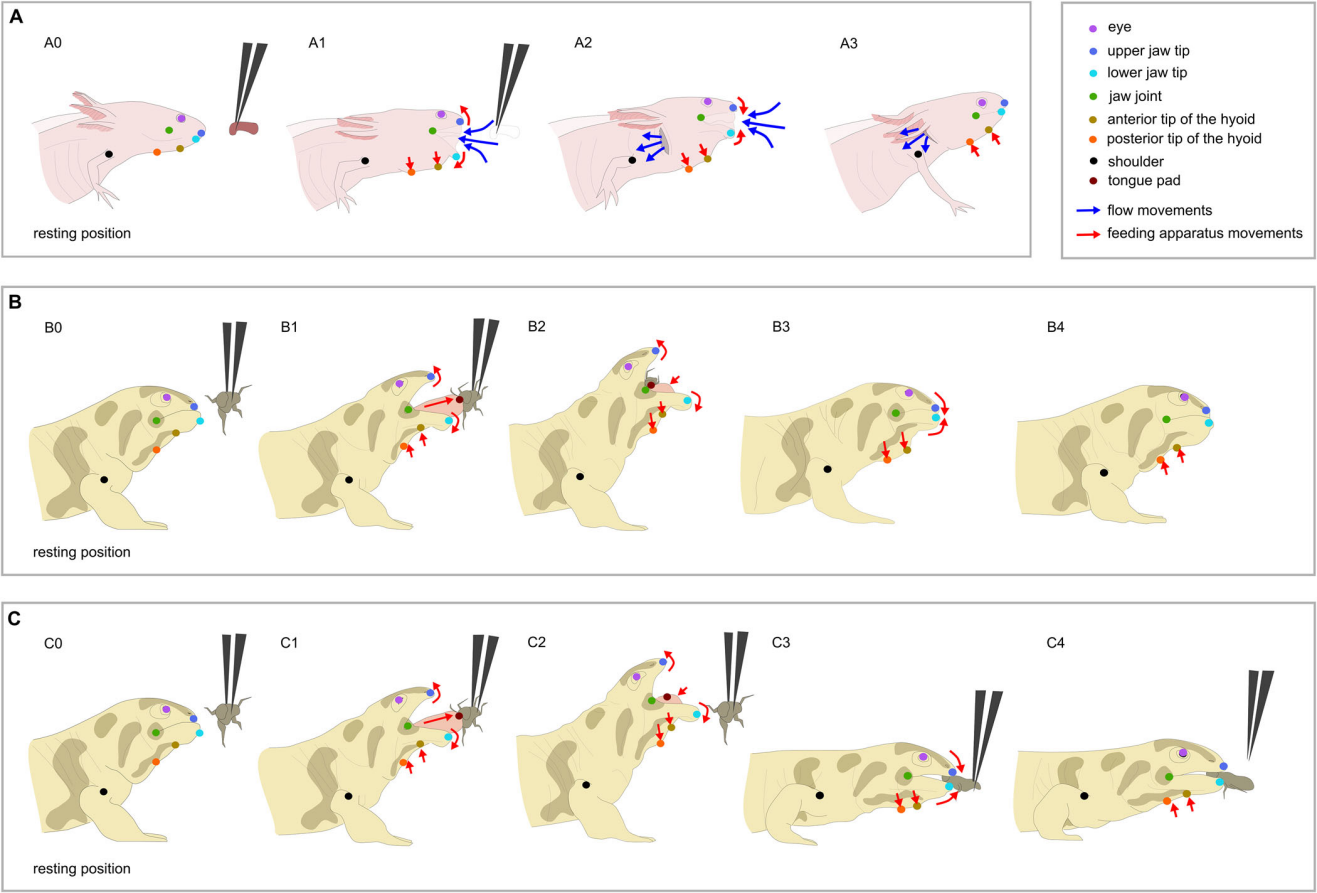
Landmarking and prey capture strategy depending on the environment. Schematic representation representing the different prey capture strategies (suction in *Ambystoma mexicanum* (A), tongue prehension (B), and jaw prehension (C) in *Ambystoma mavortium* and *Ambystoma tigrinum*) and the landmarks used to quantify the kinematics. During suction feeding (A), mouth opening quasi simultaneously followed by hyoid apparatus depression, create a flow of water which carries the prey inside the mouth (A1). The mouth starts to close before maximum hyoid depression is reached, so the flow of water keeps entering the mouth (A2). At the same time, the gill slits open and the flow is expelled at the back of the head in a unidirectional flow (A2). When the mouth is closed, the hyoid apparatus elevates to its resting position, expelling the rest of water through the gill slits. During tongue prehension, the tongue pad is protracted and contacts the prey (B1). Prey is carried by the tongue into the mouth (B2). During retraction of tongue, the gape increases so the tongue and the prey can be engulfed (B2). Jaw prehension (C) starts the same way (C1), but the tongue fails to grasp the prey (C3). It is the jaws that ensure the capture by closing themselves onto the prey (C3). In *A. mavortium* and *A. tigrinum*, hyoid apparatus and tongue movements are linked. The hyoid elevates when tongue is protracted (B1 and C1), depresses when tongue is retracted (B2, B3, C2, and C3), and elevates again when the tongue returns to its resting position (B4 and C4).

In this context, we investigated the impact of prey presentation methods on the feeding behavior of salamanders. Feeding behavior in salamanders provides an excellent framework as it is relatively well‐described and studied. Three main feeding behaviors have been described in salamanders: (i) suction feeding in water, a mechanism that relies on a fast expansion of the buccal cavity that generates a flow which carries the prey inside the mouth (Heiss and De Vylder 2016; Figure 1A; Supplementary Movie 1); (ii) tongue prehension, or (iii) jaw prehension on land, depending on whether the prey is grasped by the sticky pad of the tongue or by the jaws (Heiss and De Vylder 2016; Figure 1B,C; Supplementary Movie 2; Supplementary Movie 3; Supplementary Movie 4). Many authors have explored and compared these three feeding strategies using a similar method consisting of filming salamanders capturing prey in lateral view with high‐speed video cameras (Beneski et al. 1995; Deban and Marks 2002; Deban and O'Reilly 2005; Deban 1997; Deban et al. 2007; Deban and Richardson 2011; Deban et al. 2020; Erdman and Cundall 1984; Findeis and Bemis 1990; Heiss and De Vylder 2016; Heiss, Natchev et al. 2013; Heiss, Aerts et al. 2013; Heiss et al. 2015; Heiss and Grell 2019; Larsen and Beneski 1988; Larsen and Guthrie 1975; Larsen et al. 1989; Elwood and Cundall 1994; Lukanov et al. 2016; Miller and Larsen 1990; Reilly and Lauder 1989a; Reilly and Lauder 1992; Reilly 1995; Reilly 1996; Rull‐Garza et al. 2020; Shaffer and Lauder 1985; Stinson and Deban 2017a). However, when reviewing the experimental protocols of these studies, some details differ. For instance, depending on several factors such as the prey available in the lab, the diet, the size, the age, or the living environment of the salamanders studied, the type of prey varies from insects to worms, fish, or even shrimps. Moreover, the prey presentation method differs from one study to another. Indeed, in most studies, the prey is placed on the substrate in front of the animal (Deban 1997; Deban et al. 2007; Deban and Richardson 2011; Deban et al. 2020; Erdman and Cundall 1984; Findeis and Bemis 1990; Heiss and De Vylder 2016; Heiss and Grell 2019; Larsen and Guthrie 1975; Lukanov et al. 2016) or alternatively suspended by tweezers (Beneski et al. 1995; Larsen et al. 1989; Reilly and Lauder 1989a; Reilly and Lauder 1992). Some studies mix both of these prey presentation methods (Deban and Marks 2002; Heiss, Aerts et al. 2013; Heiss et al. 2015; Larsen and Beneski 1988; Miller and Larsen 1990). Finally, in a few studies, the prey is suspended by means of a thread (Erdman and Cundall 1984; Heiss, Natchev et al. 2013), or it is not specified how the prey is presented (Deban and O'Reilly 2005; Elwood and Cundall 1994; Reilly 1995; Reilly 1996; Stinson and Deban 2017a). While previous studies have examined how prey size, elusiveness, and movement affect aquatic and terrestrial feeding in salamanders (Deban 1997; Maglia and Pyles 1995; Luthardt, 2010; Reilly and Lauder 1989b; Rull‐Garza et al. 2020), the effect of the conditions in which the preys are presented has, to the best of our knowledge, never been assessed.

In this study, we aim to test how the conditions in which preys are presented influence feeding kinematics in aquatic and in terrestrial environments. To do so, we collected feeding sequences from aquatic (axolotls, *Ambystoma mexicanum* [Shaw and Nodder 1798]) and terrestrial salamanders (tiger salamanders, *Ambystoma tigrinum* [Green, 1825] and barred tiger salamanders, *Ambystoma mavortium* Baird 1850). Two prey presentation methods were chosen: (1) prey placed on the substrate, (2) prey suspended by tweezers. We hypothesize that the conditions under which the prey is presented will have an impact on the feeding kinematics as individuals will have to exert more force to pull the prey when it is held by the tweezers. However, in line with previous observations (Dawkins 2007; Moyaho and Beristain‐Castillo 2019; Lauder and Shaffer 1986), we expect feeding kinematics to be more affected by interindividual variation and by differences between feeding on land and in water medium. Thus, our study has three main objectives: (1) to test the effect of prey presentation method feeding kinematics, (2) to assess if individual variation overrides the impact of prey presentation method, and (3) to evaluate whether differences between aquatic and terrestrial feeding are more pronounced than those due to prey presentation method alone.

## 2 Materials and Methods

### 2.1 Material

All the specimens belong to Ambystomatidae and came from breeding colonies in French laboratories (UMR 7179, CNRS‐MNHN, Paris, France). For the kinematic analyses in aquatic conditions, five sexually immature specimens of axolotls, *Ambystoma mexicanum* (Shaw and Nodder 1798) were used. *Ambystoma mexicanum* is an obligate paedomorphic species, meaning the individuals retain larval characteristics and stay aquatic for their entire life. They use suction feeding to capture their prey. For the kinematic analyses in terrestrial conditions, two adult specimens of the Western tiger salamander, *Ambystoma mavortium* Baird, 1850, and three specimens of the Eastern tiger salamander, *Ambystoma tigrinum* (Green 1825), were used. *Ambystoma tigrinum* and *A. mavortium* are biphasic species, with eggs that hatch into aquatic larvae which, after a period of growth, metamorphose into terrestrial adults. *Ambystoma mavortium* and *A. tigrinum* were considered the same species until Shaffer and McKnight (1996) provided molecular phylogenetic data proving they should be regarded as distinct species. However, despite these molecular differences, they are cryptic species meaning they share the same morphology. This is why we combined them as one group in this study. They feed using tongue or jaw prehension. Specimens of visually similar size ranges were selected within each group. We subsequently measured a proxy for size for each individual, provided in Table 1. Sex was not controlled for because there is no sexual dimorphism in head morphology within the Ambystomatidae family.

**Table 1:** Sample information. The table details the number of videos per individual and per group depending on how the prey was presented. The size of each individual (snout‐vent length, SVL, or eye to nose distance) is also given. Quantification of the SVL consisted in measuring the SVL on one frame of each video with imageJ (Schneider et al. 2012). The SVL attributed to a given individual corresponded to the average of the SVL measurements from the different videos of that individual.

	On the substrate	Suspended by tweezer	Size
Aquatic data set	23	31	SVL (cm)
A1	5	6	6.83
A2	5	6	6.84
A3	4	7	6.49
A4	5	6	6.53
A5	4	6	7.72
Terrestrial data set	20	31	SVL (cm)
T1	3	3	12.46
T2	5	7	14.03
T3	4	6	13.57
T4	4	6	13.88
T5	4	9	13.50

*Note:* A1‐5 are the five specimens of *Ambystoma mexicanum*; T1‐2 are the two specimens of *Ambystoma mavortium*; T3‐5 are the three specimens of *Ambystoma tigrinum*.

Abbreviation: SVL, snout‐vent length.

### 2.2 Experimental Design

Specimens were placed in a transparent glass tank with a background consisting of a 0.5 × 0.5 cm checkerboard as a scale. For aquatic specimens, the tank was filled with water at room temperature (18°C). Two infrared lights (dedolight LEDs) were used to record videos under low light conditions without disturbing the animals. Feeding sequences were recorded in lateral view using a high‐speed Phantom Miro R 311 camera, set at 1000 fps, with a Nikon 50 mm lens, and connected to a computer with the PCC (Phantom Camera Control) software (version 3.6). Videos were registered in “.cine” format.

For aquatic as well as terrestrial specimens, two prey presentation methods were tested. Condition 1: the prey was presented on the substrate in front of the animal. Condition 2: the prey was above ground, suspended by a pair of tweezers in front of the animal. As prey capture is highly stimulated by prey movements in salamanders, we only used live prey. For aquatic specimens, we used pieces of earthworms of about 2–3 cm long. Preys used for terrestrial specimens were crickets of about 1–2 cm long. To minimize differences in prey mobility between these two prey types, crickets were immobilized before being laid on the substrate. This procedure allowed the crickets to retain some limb and antenna movement but prevented them from running or jumping.

Specimens were filmed each day during two consecutive weeks to obtain at least three good lateral view videos for each prey presentation method. When a salamander did not feed when tested, it was put back in its tank of origin and tested again the day after. Only feeding sequences in which the salamander remained in lateral view and in which prey was successfully captured were used for kinematic analyses. The number of videos per individual and prey presentation method is summarized in Table 1.

### 2.3 Data Extraction

Videos were cropped to keep only the feeding sequences starting with the opening of the mouth and ending with the return of the hyoid apparatus to its resting position. Videos were converted in “.mp4” format for compatibility with Deeplabcut (DLC; Nath et al. 2019; Mathis et al. 2018). This software is an efficient method for 2D markerless pose estimation based on transfer learning with deep neural networks (Nath et al. 2019; Mathis et al. 2018). Aquatic feeding and terrestrial feeding were treated in two distinct DLC projects but the methodology used was generally the same. In both cases, 50 frames were extracted per video. On these extracted frames, several markers were manually placed on the different body parts of each individual: the eye (e), the upper jaw tip (uj), the lower jaw tip (lj), the jaw joint (jj), the anterior tip of the hyoid (hd1), the posterior tip of the hyoid (hd2) and the shoulder (sh), and, for the terrestrial individuals only, also the tongue pad (tg) (Figure 1). A neural network was trained through 500,000 iterations using a batch size = 8. The landmarking was verified using the labeled video produced after the training. If the landmarking was not accurate, new images were extracted and labeled, and new iterations were run to improve the training. This process was repeated until predictions were sufficiently accurate (landmark likelihood > 0.9; labeled videos).

At the end of this process, .csv fields containing the 2D coordinates of the landmarks were extracted for each video. Next, R (version 4.4.2; R Core Team 2024 —https://www.R-project.org/) was used to convert times from frame number to seconds, and distances from pixels to centimeters, and to calculate variables concerning the movement of the jaws and hyoid apparatus. All the curves of x and y coordinates changes over time were smoothed with a cubic smoothing spline using the “smooth.spline” function (package stats v4.4.2) and 80 degrees of freedom. From the smoothed (x, y) coordinates, the following primary kinematic variables were calculated frame by frame: the distance between the tips of the upper and lower jaws, the distance between the hyoid markers and the jaw joint, the speed and acceleration of mouth and hyoid movements, the angle formed between the upper jaw, the jaw joint, and the lower jaw, and the angle formed between the upper jaw, eye, and shoulder. For the terrestrial individuals, we also calculated the distance between the tongue pad and the jaw joint as well as speed and acceleration of the tongue movements.

Distances were calculated using the formula: $d\mathrm{AB}=\sqrt{{\left(\mathrm{xA}-\mathrm{xB}\right)}^{2}+{\left(\mathrm{yA}-\mathrm{yB}\right)}^{2}}$, *d*AB being the distance between points A and B. Speeds and accelerations were calculated using the “diff” function (package stats v4.4.2), with speeds derived from distance over time and accelerations derived from speed over time. Angles were calculated by applying Al‐Kashi's theorem, according to which: $\hat{\mathrm{ABC}}=\mathrm{arcos}(\frac{d{\mathrm{BC}}^{2}+d{\mathrm{AB}}^{2}-d{\mathrm{AC}}^{2}}{2*d\mathrm{BC}*d\mathrm{AB}})$ with *d*BC, *d*AB, and *d*AC being respectively the distance between points B and C, A and B, A and C. Radians were converted to degrees by multiplying by 180°/π.

### 2.4 Kinematic Variables

From the primary variables, 25 kinematic variables common to both terrestrial and aquatic species were extracted. For the terrestrial individuals, seven additional kinematic variables relative to the tongue movement were calculated. The feeding kinematic variables are listed below.

- Kinematic variables related to mouth movement (common to terrestrial and aquatic animals):
  MG, maximum gape distance;
  TMG, time to maximum gape distance;
  MGA, maximum gape angle;
  DG, duration of the gape cycle from opening to closing of the mouth;
  MSGO, maximum speed of jaw opening;
  MAGO, maximum acceleration of jaw opening;
  MSGC, maximum speed of jaw closing;
  MAGC, maximum acceleration of jaw closing;
- Kinematic variables related to the movement of the anterior part of the hyoid (hd1; common to terrestrial and aquatic):
  Mhd1, maximum hd1 depression;
  TMhd1, time to maximum hd1 depression;
  Dhd1, duration of hd1 cycle;
  MSDhd1, maximum speed of hd1 depression;
  MADhd1, maximum acceleration of hd1 depression;
  MSEhd1, maximum speed of hd1 elevation;
  MAEhd1, maximum acceleration of hd1 elevation;
- Kinematic variables related to the movement of the posterior part of the hyoid (hd2; common to terrestrial and aquatic):
  Mhd2, maximum hd2 depression;
  TMhd2, time to maximum hd2 depression;
  Dhd2, duration of hd2 cycle;
  MSDhd2, maximum speed of hd2 depression;
  MADhd2, maximum acceleration of hd2 depression;
  MSEhd2, maximum speed of hd2 elevation;
  MAEhd2, maximum acceleration of hd2 elevation;
- Kinematic variables relative to the overall prey capture event (common to terrestrial and aquatic):
  MHA, maximum head angle during prey capture;
  TMHA, time to maximum head angle;
  PCD, prey capture duration from the initial opening of the mouth (t0) to the return of the hyoid apparatus to its initial position;
- Kinematic variables relative to tongue movement (only terrestrial):
  MTgP, maximum tongue protraction which corresponds to maximal distance between the jaw joint and the tongue pad;
  TMTgP, time to maximum tongue protraction;
  TgD, tongue movement duration from the beginning of protraction to return to the buccal floor;
  MSTgP, maximum speed of tongue protraction;
  MATgP, maximum acceleration of tongue protraction;
  MSTgR, maximum speed of tongue retraction;
  MATgR, maximum acceleration of tongue retraction.

For terrestrial feeding, it is important to notice that in Caudata, tongue movements are linked to the hyobranchial apparatus movements (Reilly and Lauder 1989a; Stinson and Deban 2017b; Özeti et al. 1969; Wake and Deban 2000; Beneski et al. 1995; Findeis and Bemis 1990; Miller and Larsen 1990). For this reason, we consider only the depression and the elevation of the hyoid occurring after maximum protraction of the tongue. The hyoid depresses when the tongue is retracted in direction to the throat then the hyoid elevates when the tongue returns to its resting position (Figure 1B,C).

### 2.5 Head Position and Snout‐Prey Distance at the Onset of the Strike

Head angle at strike onset (HA0) was also recorded from the primary variables for both aquatic and terrestrial data sets. To assess whether HA0 was influenced by the prey presentation method, we fitted linear mixed models using the “lmer” function from the lmerTest package (v3.1.3; Kuznetsova et al. 2017), treating individual identity as a random effect. We then used the “emmeans” function from the emmeans package to compute pairwise contrasts between the two prey presentation methods. If a significant effect of prey presentation on HA0 was detected, we visualized the differences using the “ggboxplot” function from the ggpubr package (built on ggplot2, v3.5.1; Wickham 2016).

Animals were free to choose their striking distance, as this variability reflects natural behavior. Consequently, snout‐prey distance at strike onset (SPD0) was also measured using imageJ (Schneider et al. 2012). R (version 4.4.2; R Core Team 2024) was used to obtain the mean, standard deviation, and minimum and maximum values of SPD0.

### 2.6 Statistical Analyses of Feeding Kinematics

#### 2.6.1 Testing the Impact of Prey Presentation Method and Individual on Feeding Kinematics

For both the aquatic and terrestrial data set, the number of kinematics variables (25 for suction feeding and 32 for terrestrial feeding) approaches the number of observations per condition (Aquatic: 31 videos with tweezer and 23 without; Terrestrial: 31 videos tweezer and 20 without; Table 1). To avoid variable reduction, each data set was fitted to a linear model using function “mvols” of mvMORPH package (v 1.2.1), which is suitable for high‐dimensional data sets (Clavel and Morlon 2020). Models fitted took into account the interaction term between the prey presentation method and the individual (prey_presentation_method x ind).

Two methods were tested when fitting the model: the penalized log‐likelihood method “PL‐LOOCV” and the log‐likelihood method “LL.” To know which method fitted the data best, the generalized information criteria (GIC) of each method were compared (see Supplementary Table S1). The method fitting the best ‐which corresponded to the model with the smaller GIC‐ was selected for the following analyses. For the aquatic data set, the method “PL‐LOOCV” was selected. For the terrestrial data set, the method “LL” fitted best (Supplementary Table S1). Once the best method selected, the multinormality of the model residuals was verified using the function “mvqqplot” of the mvMORPH package. For each data set a type two Multivariate analysis of variance (Type II MANOVA), taking into account the interaction between the prey presentation method and the individual effect was then performed using Pillai test with the function “manova.gls” in the mvMORPH package. As the aquatic data set was fitted with the method PL‐LOOCV, we used a non‐parametric permutation‐based type II MANOVA with 5000 permutations to assess significance. For the tongue prehension model, fitted with the LL method, we used a parametric type II MANOVA.

#### 2.6.2 Testing Individual Differences for Models Considering the Interaction Between the Prey Presentation Method and the Individuals

To explore individual differences in prey capture, post‐hoc tests were realized on MANOVA output, using the function “pairwise.glh” in mvMORPH. For the aquatic data set, the pairwise comparison was performed using non‐parametric tests with 5000 permutations.

#### 2.6.3 Testing the Impact of Prey Presentation Method on Suction Feeding Kinematics

**Figure 2:**
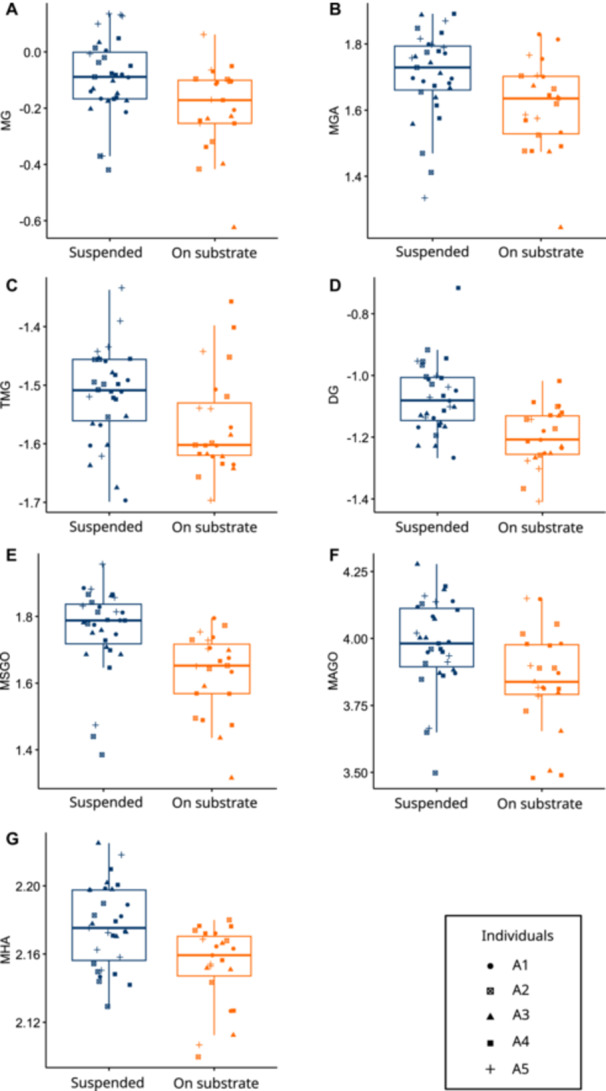
Boxplot of the kinematic variables that showed significant differences depending on the prey presentation methods. The kinematic variables concerned are: (A) MG, Maximum gape distance; (B) MGA, maximum gape angle; (C) TMG, Time to maximum gape distance; (D) DG, Duration of the gape cycle from opening to closing of the mouth; (E) MSGO, Maximum speed of jaw opening; (F) MAGO, Maximum acceleration of jaw opening; (G) MHA, Maximum head angle during prey capture.

For the aquatic data set, the type II MANOVA detected an impact of the prey presentation method. To find which feeding kinematic variables were impacted, we performed separate analyses on each of them using linear mixed models where the individuals were treated as random effects using “lmer” function of lmerTest package (v3.1.3; Kuznetsova et al. 2017). The contrast between the two prey presentation methods was obtained using the “emmeans” function of the emmeans package. The two prey presentation methods are summarized using boxplots for each kinematic variable that was significantly impacted using the “ggboxplot” function of the package ggplot2 (v3.5.1; Wickham 2016) (Figure 2).

#### 2.6.4 Testing the Impact of Prey Capture Type on Terrestrial Feeding Kinematics

We also tested whether the prey capture type impacts terrestrial feeding kinematics. Terrestrial salamanders can use tongue or jaw prehension. The capture type was defined as “jaw prehension” when the prey capture was ensnared by the jaws rather than by the tongue. In our data set, “jaw prehension” always occurred when the tongue failed to grasp the prey. In these cases, the individuals still managed to capture the prey with the jaws when closing them following the tongue failure. Jaw prehension after tongue failure was previously reported by Wake and Deban (2020). All the videos of terrestrial feeding were visualized and prey capture type (tongue prehension or jaw prehension) was noted (Table 2). As before, the terrestrial data set was fitted to a linear model using “mvols” of mvMORPH package (Clavel and Morlon 2020), but this time the model took into account the interaction terms between prey capture type (jaw or tongue prehension) and the individual effect (prey_capture_type x ind). The method selected was “LL” (according to the GIC). A type II MANOVA was performed using the function “manova.gls” with a Pillai test in the mvMORPH package. As an individual impact was found, a pairwise comparison was performed using the function “pairwise.glh” of mvMORPH package. As an impact of prey capture was also detected, each kinematic variable was fitted to a linear mixed model taking into account the prey capture type and with individual implemented as a random effect. Boxplots comparing the two conditions are provided for the kinematic variables that are significantly impacted, using the ggboxplot function of package ggplot2.

**Table 2:** Number of jaw prehension sequences relative to the total number of sequences for each terrestrial individual depending on the way the prey is presented.

	Prey suspended by tweezers	Prey on the substrate
T1	0/3	0/3
T2	2/7	1/5
T3	4/6	0/4
T4	2/6	0/4
T5	4/9	0/4
All the individuals	12/31	1/20

Abbreviations: T1‐2, two specimens of *Ambystoma mavortium*; T3‐5, three specimens of *Ambystoma tigrinum*.

#### 2.6.5 Comparing the Functional Space of Aquatic and Terrestrial Feeding

To visualize whether the differences between aquatic and terrestrial species were greater than the differences due to prey presentation methods, a new data set gathering the terrestrial and aquatic values for the 25 common feeding kinematic variables was created. A Principal Component Analysis (PCA) using the correlation matrix to account for correlations among variables, was performed to visualize the kinematic space for aquatic and terrestrial prey capture events using the “prcomp” function (package stats v4.4.2). The two first axes were plotted using the “ggplot” function of package ggplot2. The contribution of the variables to each axis was visualized using “fviz_contrib” and “fviz_pca_var” functions of package factoextra (v1.0.7; Kassambara and Mundt 2020). The impact of the prey presentation method, of the medium, and of the interaction of both of these factors on the first axis of the PCA was tested by performing a type II ANOVA using “ANOVA” function of package car (v3.1.3, Fox and Weisberg 2019). Next, the impact of the medium on the kinematic variables that contributed mainly on axis 1 of the PCA was tested performing ANOVAs using the “aov” function of package stats (v4.4.2, R Core Team 2024).

To explore whether the variability in kinematics during terrestrial feeding was greater than that observed during aquatic feeding, a disparity analysis was conducted between terrestrial and aquatic individuals using the “dispRity.per.group” function of package dispRity (Guillerme 2018). Pairwise differences in kinematic disparity between aquatic and terrestrial individuals were assessed using Wilcoxon's test of significance with Bonferroni correction using the “test.dispRity” function of package dispRity.

All the R scripts are available (see Data availability).

### 2.7 IA Used in the Manuscript

ChatGPT (OpenAI, 2021), a language model based on the GPT (Generative Pre‐trained Transformer) architecture was used for helping to write part of the R scripts.

## 3 Results

### 3.1 Head Position and Snout Prey Distance at the Onset of the Strike

The head angle in aquatic individuals at strike onset was impacted by the prey presentation method (*DF* = 50.4, t. ratio = −4.326, *p *< 0.001) with a higher initial head angle when the prey was suspended (Supplementary Figure S1). By contrast the initial head angle of terrestrial individuals was not significantly impacted (*DF* = 47.2, t.ratio = 0.504, *p* = 0.616).

The snout prey distance in aquatic individuals ranged from 0 to 0.46 cm and was on average equal to 0.05 cm with a standard deviation of 0.11 cm. We noticed that the individuals were most of the time touching the prey with their snout before strike. Snout‐prey distance in terrestrial individuals ranged from 0 to 3.17 cm and was on average equal to 0.74 cm with a standard deviation of 0.69 cm. While aquatic individuals always moved closer to the prey before the strike, terrestrial individuals did not approach the prey when being further away; instead, they usually lunged or jumped suddenly toward the prey.

### 3.2 The Effect of Prey Presentation Methods and Individual for Models Considering the Interaction Between the Prey Presentation Method and the Individuals

For the aquatic data set, the type II MANOVA revealed an impact of prey presentation methods and individual, but no interaction between the two (Table 3). The estimated marginal means and associated contrasts revealed that prey presentation methods had an impact on the kinematic variables relative to mouth opening and head angle (Supplementary Table S3). The boxplots show that when the prey is suspended by tweezers, maximum gape distance (MG) and maximum gape angle (MGA) are larger (Figure 2A,B), the maximum gape is reached later (TMG; Figure 2C), the gape cycle lasts longer (DG; Figure 2D), the maximum speed and the maximum acceleration of mouth opening are faster (MSGO and MAGO; Figure 2E,F), and the maximum head angle (MHA) is greater (Figure 2G). For the terrestrial data set, the type II MANOVA showed an impact of the individual only (Table 3). However, for neither the aquatic nor the terrestrial data set did pairwise comparisons reveal significant differences between individuals (Supplementary Table S2).

**Table 3:** Results of the type II MANOVAs with Pillai's test assessing the influence of prey presentation method, prey capture type, and individual variation on feeding kinematics.

Model (prey presentation method × individuals)	Aquatic data set: Method PL‐LOOCV, 5000 permutations			
			Pillai	*p* value
	Prey presentation method		0.715	< 0.001*
	Individuals		1.890	0.004*
	Prey presentation method × individual		1.413	0.882
	*Terrestrial data set: Method LL*			
		*Df*	Pillai	*p* value
	Prey presentation method	1	0.870	0.109
	Individuals	4	3.244	0.012*
	Prey presentation method × individual	4	2.808	0.588

Model (prey capture type × individuals)	Terrestrial data set: Method LL			
		*Df*	Pillai	*p* value
	Prey capture type	1	0.952	0.001*
	Individuals	4	3.390	< 0.001*
	Prey capture type × individual	3	2.351	0.087

Abbreviations: *Df*, degree of freedom; LL, log‐likelihood method; PL‐LOOCV, penalized log‐likelihood method.

*Note:* Symbols: “x” Indicates interaction; “*” Denotes significant values.

### 3.3 The Effect of Prey Capture Type and Individual for the Model Considering the Interaction Between the Prey Capture Type and the Individuals During Terrestrial Feeding

**Figure 3:**
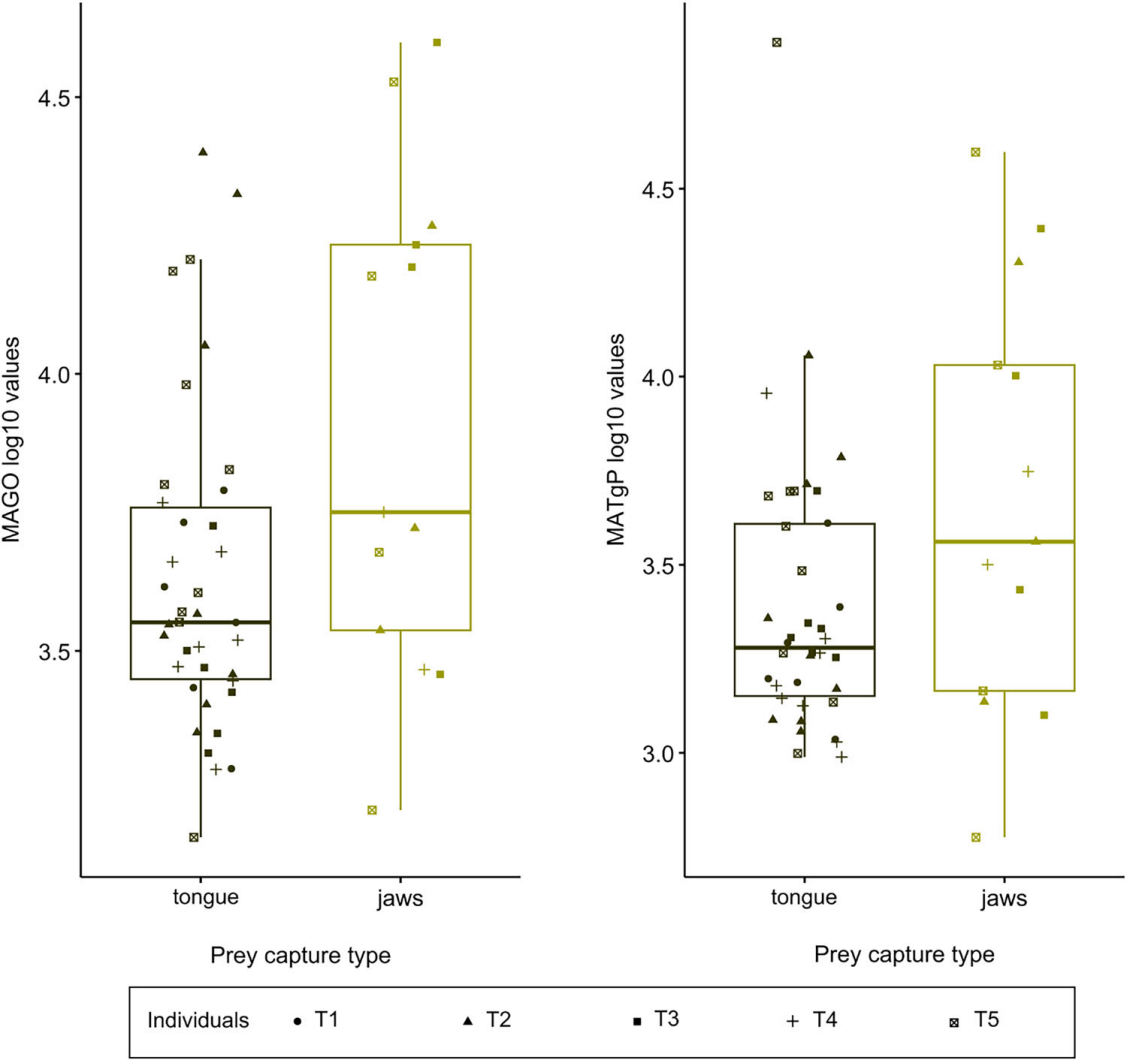
Boxplot of the kinematic variables that differ depending on the prey capture type. jaws, jaw prehension; MAGO, maximum acceleration of mouth opening; MATgP, maximum acceleration of tongue protraction; tongue, tongue prehension.

Of the 31 sequences where prey was suspended by tweezers, jaw prehension occurred 12 times (38.71%). Of the total of 20 sequences where prey was placed on the substrate, jaw prehension occurred only once (5%; see Table 2). The type II MANOVA revealed an impact of prey capture type and individual, but no interaction between these two factors (Table 3). Pairwise comparisons revealed that individual T4 differed from T2 and T5 (T4 vs. T2: *DF* = 1, Pillai = 0.940, *p* = 0.025; T4 vs. T5: *DF* = 1, Pillai = 0.940, *p* = 0.025). The estimated marginal means and associated contrasts indicated that prey capture type had an impact on the maximum acceleration of mouth opening (MAGO; *DF* = 52.6, t.ratio = 2.496; *p *= 0.016; Supplementary Table S4), and on the maximum acceleration of tongue protraction (MATgP; *DF* = 52.6, t.ratio = 2.006; *p *= 0.050; Supplementary Table S4), these two variables being higher when individual used jaw prehension (Figure 3).

### 3.4 Comparing the Functional Space of Aquatic and Terrestrial Feeding

**Figure 4:**
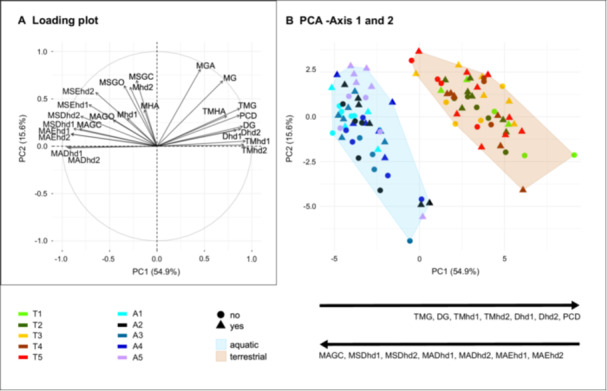
Visualization of the differences between aquatic and terrestrial feeding on the first two axes of the PCA. (A) Loading plot (B) PCA‐Axis 1 and 2. Timings and duration increase with PC1 whereas accelerations and speeds decrease with PC1. A1‐5 are the five specimens of *Ambystoma mexicanum*; T1‐2 are the two specimens of *Ambystoma mavortium*; T3‐5 are the three specimens of *Ambystoma tigrinum*. DG, Duration of the gape cycle from opening to closing of the mouth; Dhd1‐2, Duration of hd1‐2 cycle; hd1, anterior part of the hyoid; hd2, posterior part of the hyoid; MADhd1‐2, Maximum acceleration of hd1‐2 depression; MAEhd1‐2, Maximum acceleration of hd1‐2 elevation; MAGC, Maximum acceleration of jaw closing; MAGO, Maximum acceleration of jaw opening; MG, Maximum gape distance; MGA, Maximum gape angle; MHA, Maximum head angle during prey capture; Mhd1‐2, Maximum hd1‐2 depression; MSDhd1‐2, Maximum speed of hd1‐2 depression; MSEhd1‐2, Maximum speed of hd1‐2 elevation; MSGC, Maximum speed of jaw closing; MSGO, Maximum speed of jaw opening; PCD, prey capture duration; TMG, Time to maximum gape distance; TMHA, Time to the maximum head angle; TMhd1‐2, Time to maximum hd1‐2 depression.

Principal component 1 (PC1) explained 54.9% of the overall variance and separated aquatic from terrestrial feeding sequences (Figure 4). Fourteen variables contributed above the expected average contribution threshold to the PC1 axis (Supplementary Figure S2A). All the durations (DG, Dhd1, Dhd2, PCD; Figure 4) and the timings concerning the maximum excursions of the gape and hyoid apparatus (TMG, TMhd1, TMhd2; Figure 4) contributed positively to the PC1 axis. The maximum speeds during depression of the hyoid (MSDhd1 and MSDhd2) and the maximum accelerations of the closing of the mouth (MAGC) and of the depression and elevation of the hyoid apparatus (MADhd1, MADhd2, MAEhd1, MAEhd2; Figure 4), contributed negatively to the PC1 axis. Principal component 2 (PC2) explained 15.6% of the overall variance (Figure 4). Eight variables contributed above the expected average contribution threshold to the PC2 axis (Supplementary Figure S2B). The variable concerning the maximum gape (MGA, MG, TMG; Figure 4), and the maximum depression of the posterior part of the hyoid (Mhd2; Figure 4), the maximum speeds of mouth opening and closing (MSGO, MSGC; Figure 4), and the maximum speeds of the hyoid apparatus elevation (MSEhd2, MSEhd1; Figure 4) all contributed positively to the PC2 axis (Figure 4).

The type II ANOVA revealed that only the medium had an impact on the first axis of the PCA (Supplementary Table S5). All the variables contributing mainly to this axis were significantly impacted by the medium (Supplementary Table S6).

**Figure 5:**
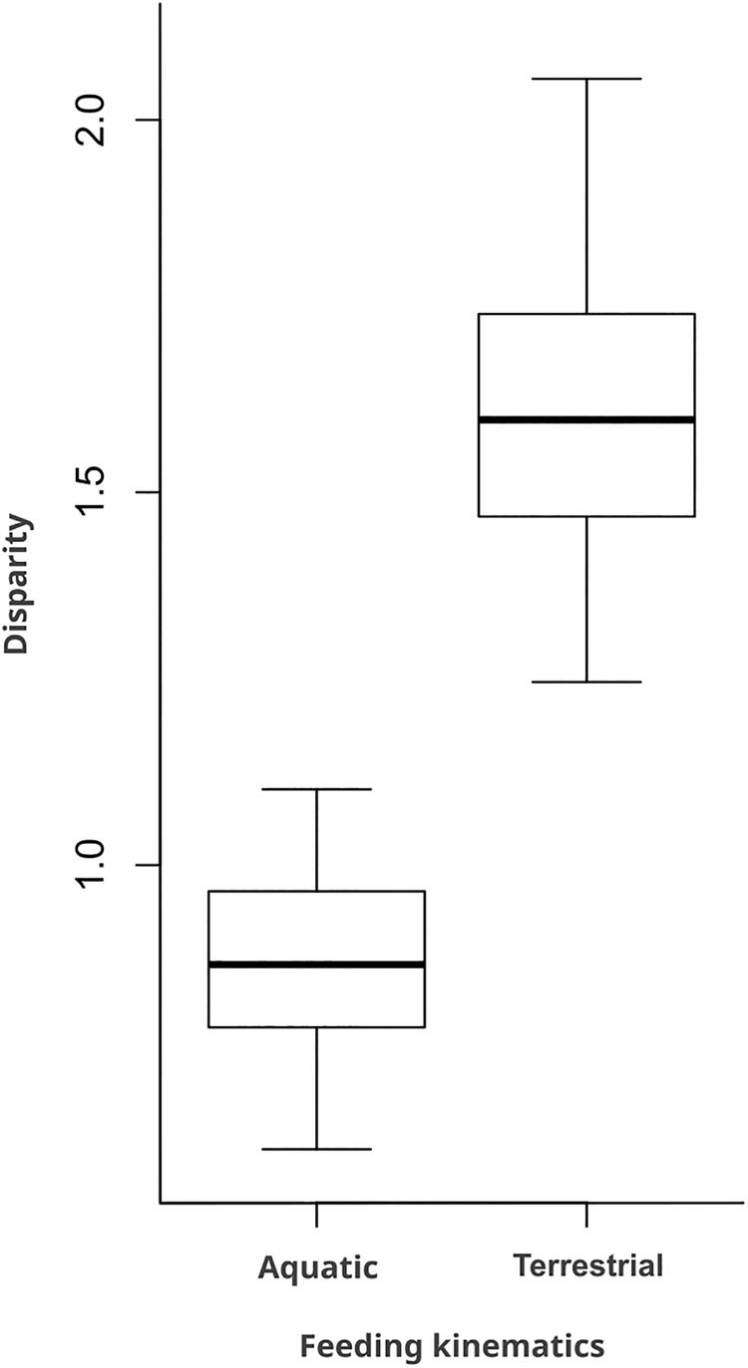
Boxplots showing the differences in disparity between aquatic and terrestrial feeding kinematics.

Disparity is lower in aquatic (0.89) than in terrestrial individuals (1.65; Figure 5). A Wilcoxon test revealed that the disparity values for each medium were significantly different (*p* < 0.001).

## 4 Discussion

### 4.1 Individual Differences Are Not Greater Than Differences Between Prey Presentation Methods

Based on the literature on different animal models (Dawkins 2007; Moyaho and Beristain‐Castillo 2019) and on the feeding behavior of *A. tigrinum* (Lauder and Shaffer 1986), we expected the feeding kinematics to be more impacted by interindividual variation than by the prey presentation methods. Our results suggest this is not the case in neither aquatic nor terrestrial environments. Indeed, the MANOVAs revealed no significant impact of the interaction term between the prey presentation method and individual (Table 3), meaning that in a given environment, all the individuals are affected the same way by the prey presentation methods. Nevertheless, the individual effect was also significant showing that individuals do differ from one another. However, the pairwise comparisons no longer indicated significant differences between individuals suggesting that the individual effect is small (Supplementary Table S2). This is also visible in the PCA where the feeding sequences of the different individuals clearly overlap in the kinematic space (Figure 4).

### 4.2 Axolotls Adapt Their Initial Position and Their Suction Feeding Kinematics Depending on Prey Presentation Methods

The way prey was presented to the axolotls significantly impacted the initial head angle as well as the kinematic variables concerning the mouth opening and the maximum head angle (Figure 2). Three main differences can be highlighted between the two prey presentation methods.

First, the most obvious difference is the position of the prey in the environment, suspended or on the substrate. Since suction force decreases rapidly with distance from the mouth (Wainwright and Day 2007), this likely explains why individuals were always extremely close to the prey before the strike (snout touching the prey or not more far than 0.46 cm). As a result, the initial head angle was also impacted with a more dorsiflexed neck position when the prey was suspended, and a more ventroflexed position when the prey was on the substrate (Supplementary Figure S1).

A second difference lies in the type of medium in contact with the prey: when the prey is suspended, it is only surrounded by water, whereas when on the substrate, it is in contact with both water and substrate. Interestingly, in a study investigating suction feeding of white‐spotted bamboo sharks (*Chiloscyllium plagiosum*, Bennett 1830), Nauwelaerts et al. (2007) compared feeding kinematics when prey was suspended in water column versus when the prey was placed on the substrate. They showed that suction feeders have to be close to their prey for the suction flow to be strong enough to successfully carry the prey inside the mouth. However, they also demonstrated that the presence of a substrate increased the efficiency of the suction flow. Indeed, when the prey is suspended and surrounded by water, suction occurs in a 3‐dimensional space, whereas when the prey is placed on the substrate, the space becomes relatively 2‐dimensional, which permits to concentrate the flow (Nauwelaerts et al. 2007). In fact, for the same suction flow exerted on the same prey, the presence of a substrate permits to double the maximum distance—between the predator's mouth and the prey—at which prey capture is successful (Nauwelaerts et al. 2007).

A third important difference between the two prey presentation methods is the fact that when placed on the substrate, the prey are free, whereas when suspended, they are held in place, pinched by the tweezer which impacts the suction feeding kinematics. A suction flow can exert three main forces on the prey: the pressure gradient force, the drag force, and the acceleration reaction (Wainwright and Day 2007). The pressure gradient force is always present and results from the expansion of the buccal cavity which propagates a pressure gradient from the lowest pressure inside the mouth to higher pressure at a distance from the mouth (Wainwright and Day 2007). It is this pressure gradient that pulls the prey toward the buccal cavity (Wainwright and Day 2007). However, the drag force, which is the force generated by the displacement of the fluid relative to the prey (Wainwright and Day 2007) and the acceleration reaction, which is the force generated when the velocity of the fluid moving relative to the prey increases over time (Day et al. 2005; Wainwright and Day 2007), are nonexistent if the prey moves like a water particle (Wainwright and Day 2007). By contrast, drag and acceleration force have an important impact when the prey is fixed (Wainwright and Day 2007). Thus, in our study, when the prey is placed on the substrate, the drag and acceleration force might be small compared to when the prey is held by tweezers. In addition, the drag force is influenced by passive characteristics of the prey such as its surface area, its shape or orientation (Nemeth 1997). Thus, the fact that a prey item is completely surrounded by water or in contact with a substrate can also have an impact on the drag force.

While comparing the two prey presentation methods in our results, prey capture seems facilitated by the presence of a substrate in part also because the prey is free. The fact that axolotls amplify their mouth opening movements, and increase the speed and acceleration of mouth opening when the prey is held by tweezers (Figure 2) may simply reflect the greater force needed to drag the prey into the mouth. Moreover, the greater maximum head angle observed (Figure 2G) is expected, as it reflects the same pattern seen in the initial head angle, resulting from the prey being positioned above the animal. However, the interpretation of the observed kinematic differences can be further refined when considering the simulations of Wainwright and Day (2007). These simulations demonstrated that the pressure gradient force is the main force exerted on the prey during suction (Wainwright and Day 2007) and showed there are two ways to increase the pressure gradient force: a spatial and a temporal way. The spatial way consists in reducing the size of mouth opening, which decreases the magnitude of forces far from the mouth, but drastically increases the imparted forces near the mouth (Wainwright and Day 2007). The temporal way, consists in increasing the rate at which peak flow velocity is achieved (Wainwright and Day 2007). In our results, we found that mouth is opened wider when the prey is suspended by tweezers compared to when it is placed on the substrate (Figure 2A,B). Thus, a narrower mouth opening when the prey is on the substrate may be linked to the spatial way of increasing the strength of the pressure gradient. Nevertheless, a narrower mouth opening means the magnitude of forces decreases quickly far from the mouth. Yet, this is likely compensated by the presence of the substrate, which permits to double the distance at which prey can be successfully captured (Nauwelaerts et al. 2007). By contrast, the fact that the maximum speed and acceleration of mouth opening are faster when the prey is suspended (Figure 2E,F) can be interpreted as a temporal way to increase the pressure gradient force. Finally, differences in timing and duration (TMG and DG; Figure 2C,D) are likely linked to the amplitude of the mouth opening: maximum gape is reached later when the gape is wider, and as a result the gape cycle duration also lasts longer. Thus, the differences in kinematics observed here may reflect two adaptative behaviors used to increase the pressure gradient force depending on the way the prey is presented.

### 4.3 Prey Presentation Method Does Not Impact the Terrestrial Initial Position and Feeding Kinematics

Contrary to what was observed during suction feeding, in terrestrial feeding we did not detect differences in initial head angle nor in prey capture kinematics (Table 3) depending on the prey presentation method. However, it may have an indirect impact on prey capture type. Indeed, jaw prehension—which occurred only when the tongue failed to successfully capture the prey and appeared to result from higher mouth opening acceleration and tongue protraction acceleration (Figure 3)—potentially reflects a miscalculation by the predator, which happened mostly when the prey was suspended (Table 2).

### 4.4 Kinematics Differ Based on the Medium

Aquatic and terrestrial individuals were not fed the same prey type. Although cricket mobility was restricted to approximate that of a piece of earthworm, differences in size and shape remained, which may have influenced the comparison of aquatic and terrestrial feeding kinematics. Nevertheless, the most important difference lies in the medium in which prey capture occurred. Indeed, water and air have radically different properties, water being 60 times more viscous and 830 times denser than air (Denny 1990). Suction feeding is therefore only adapted to the aquatic environment, as it takes advantage of the viscosity of water to suck prey into the animal's mouth. Tongue and jaw prehension, on the other hand, are adapted to the terrestrial environment.

Our results showed that buccal expansion during suction feeding is extremely rapid in the axolotls. As such, they likely cannot rely on sensory feedback to modulate the movement during buccal expansion. This means that they have to evaluate the position of the prey and the elements that may affect capture success. This is typical of a feedforward mechanism (Deban et al. 2001). All this is consistent with the fact they adjust their initial head angle and have a consistent snout prey distance, as well as with the fact they adjust their feeding kinematics depending on the prey presentation method.

In contrast, during terrestrial feeding, movements are significantly slower (Figure 4; Supplementary Table S6). Salamanders can rely on feedback from their tongue pad to modulate the feeding kinematics. They can detect when the tongue makes contact with the prey and whether the prey remains attached to the tongue pad during retraction. The fact they switch to a jaw prehension mode when tongue prehension is not successful particularly highlights this feedback mechanism.

The reduced constraints of terrestrial feeding, compared to aquatic feeding, are evident in several ways. First, the initial head angle remains unaffected by the prey presentation method. Second, the snout–prey distance shows high variability. Finally, the feeding kinematic disparity values indicate that, when tongue movements are excluded, terrestrial feeding displays greater kinematic diversity than aquatic feeding (Figure 5). Finally, differences in control strategies, feedforward versus feedback, may explain why the prey presentation method selectively affects suction feeding kinematics.

## 5 Conclusion

Suction feeding was impacted by changes in prey presentation methods, in contrast to feeding on land. The most probable causes are the differences in control strategies used, linked to the physical properties of the environment. In the aquatic medium, feeding likely relies on feedforward mechanism and as such is particularly sensitive to the changes in hydrodynamics caused by the modifications of the prey position. In contrast, terrestrial feeding depends on feedback and is less constrained which may explain the lack of differences in kinematics due to prey presentation methods. However, when aquatic and terrestrial data sets are combined, the effect of prey presentation method is overridden by the more substantial differences between aquatic and terrestrial feeding strategies which suggests that, depending on the level of analysis and the question being assessed, the importance of accounting for prey presentation method is context‐dependent. Nonetheless, in contexts where the combination of different prey presentation methods may bias the interpretation of prey capture, it may be preferable to homogenize prey presentation method, or to account for it using appropriate statistics. Measuring the interindividual and inter‐treatment effects is desirable when using data that are not homogenous.

## Conflicts of Interest

The authors declare no conflicts of interest.

## Supporting information


**Supplementary figure S1:** The prey presentation method significantly impacts the initial head angle of *A. mexicanum*. **Supplementary figure S2:** Contribution of the variables to the main PC axis. **Supplementary table S1:** Generalized information criterion (GIC) for the different methods. **Supplementary table S2:** Pairwise comparison between individuals. **Supplementary table S3:** Results of the contrasts between kinematic variables when the prey is on the substrate versus when the prey is suspended by tweezers in the aquatic data set. **Supplementary table S4:** Results of the contrasts between jaw and tongue prehension kinematics in the terrestrial data set. **Supplementary table S5:** Results of the type II ANOVA when testing for the impact of prey presentation method and medium on the first axis of the PCA. **Supplementary table S6:** Results of the analysis of variance testing the impact of the medium on the main feeding kinematics contributing to the first axis of the PCA. **Supplementary Movie 1:** Example of a typical suction feeding sequence. **Supplementary Movie 2:** Example of a tongue prehension sequence. **Supplementary Movie 3:** Example of a jaw prehension sequence when the prey is held by tweezers. **Supplementary Movie 4:** Example of a jaw prehension sequence when prey is on the substrate.

SM1_gold1_free_prey_seq1_filtered_labeled.

SM2_A_tigrinum_ind1_free_prey_seq4_filtered_labeled.

SM3_A_mavortium_ind3_tweezer_seq2_filtered_labeled.

SM4_A_mavortium_ind3_free_prey_seq6_filtered_labeled.

## Data Availability

All scripts used for kinematic variables extraction and statistical analysis are publicly available in the GitHub repository titled Prey_presentation_methods_impact: https://github.com/IToussaint-Larde/Prey_presentation_methods_impact. Raw Data (the.csv file containing time and space scales for each video as well as all the.csv files containing the 2D coordinates of the landmarks over time built by deeplabcut) is available in the GitHub repository titled Prey_presentation_methods_impact: https://github.com/IToussaint-Larde/Prey_presentation_methods_impact. Video data are available upon request.
