# Supplementary material for: Testing the Impact of Prey Presentation Method on the Feeding Kinematics of Terrestrial and Aquatic Ambystomatidae
Source: J Exp Zool A Ecol Integr Physiol. 2025 Aug 20;343(10):1150–63. doi: 10.1002/jez.70028 (PMC12604684; doi:10.1002/jez.70028)
Supplement: Supplementary file 1 — Supplementary figure S1: The prey presentation method significantly impacts the initial head angle of A. mexicanum. Supplementary figure S2: Contribution of the variables to the main PC axis. Supplementary table S1: Generalized information criterion (GIC) for the different methods. Supplementary table S2: Pairwise comparison between individuals. Supplementary table S3: Results of the contrasts between kinematic variables when the prey is on the substrate versus when the prey is suspended by tweezers in the aquatic data set. Supplementary table S4: Results of the contrasts between jaw and tongue prehension kinematics in the terrestrial data set. Supplementary table S5: Results of the type II ANOVA when testing for the impact of prey presentation method and medium on the first axis of the PCA. Supplementary table S6: Results of the analysis of variance testing the impact of the medium on the main feeding kinematics contributing to the first axis of the PCA. Supplementary Movie 1: Example of a typical suction feeding sequence. Supplementary Movie 2: Example of a tongue prehension sequence. Supplementary Movie 3: Example of a jaw prehension sequence when the prey is held by tweezers. Supplementary Movie 4: Example of a jaw prehension sequence when prey is on the substrate. [file JEZ-343-1150-s005.docx]

**Supporting information**

**Supplementary table S1. Generalized information criterion (GIC) for the different methods.** Symbols: ‘x’ Indicates interaction; bold character indicates the best GIC. Abbreviations: PL-LOOCV, penalized log-likelihood method; LL, log-likelihood method; GIC, Generalize information criterion.

| Dataset | Interaction | PL-LOOCV GIC | LL GIC |
| --- | --- | --- | --- |
| aquatic | Prey offering condition x individual | **-1285.076** | -1206.334 |
| terrestrial | Prey offering condition x individual | -895.9968 | **-1148.652** |
| terrestrial | Prey capture type x individual | -989.9443 | **-1327.426** |

**Supplementary table S2. Pairwise comparison between individuals.** A1-5 are the five specimens of *Ambystoma mexicanum*; T1-2 are the two specimens of *Ambystoma mavortium*; T3-5 are the three specimens of *Ambystoma tigrinum*. Symbols: ‘x’ Indicates interaction. ‘*’ Denotes significant value. Abbreviation: Df, degree of freedom.

| Model (prey offering conditions x individual) | Aquatic dataset | | | |
| --- | --- | --- | --- | --- |
|  |  |  | Pillai | *P* |
|  | A1 - A3 |  | 0.340 | 1.000 |
|  | A1 - A4 |  | 0.432 | 1.000 |
|  | A1 - A5 |  | 0.521 | 1.000 |
|  | A1 - A2 |  | 0.284 | 1.000 |
|  | A3 - A4 |  | 0.362 | 1.000 |
|  | A3 - A5 |  | 0.529 | 1.000 |
|  | A3 - A2 |  | 0.300 | 1.000 |
|  | A4 - A5 |  | 0.546 | 0.768 |
|  | A4 - A2 |  | 0.366 | 1.000 |
|  | A5 - A2 |  | 0.504 | 1.000 |
|  |  |  |  |  |
|  | Terrestrial dataset | | | |
|  |  | Df | Pillai | *P* |
|  | T1 - T2 | 1 | 0.885 | 0.503 |
|  | T1 - T3 | 1 | 0.762 | 1.000 |
|  | T1 – T4 | 1 | 0.939 | 0.061 |
|  | T1 - T5 | 1 | 0.841 | 1.000 |
|  | T2 - T3 | 1 | 0.830 | 1.000 |
|  | T2 - T4 | 1 | 0.851 | 0.998 |
|  | T2 - T5 | 1 | 0.728 | 1.000 |
|  | T3 - T4 | 1 | 0.925 | 0.131 |
|  | T3 - T5 | 1 | 0.761 | 1.000 |
|  | T4 - T5 | 1 | 0.891 | 0.478 |
|  |  |  |  |  |
| Model (prey capture type x individual) | Terrestrial dataset | | | |
|  |  | Df | Pillai | *P* |
|  | T1 - T2 | 1 | 0.647 | 1.000 |
|  | T1 – T3 | 1 | 0.604 | 1.000 |
|  | T1 - T4 | 1 | 0.912 | 0.105 |
|  | T1 - T5 | 1 | 0.865 | 0.499 |
|  | T2 - T3 | 1 | 0.812 | 0.995 |
|  | T2 - T4 | 1 | 0.940 | 0.025* |
|  | T2 - T5 | 1 | 0.839 | 0.760 |
|  | T3 - T4 | 1 | 0.914 | 0.104 |
|  | T3 - T5 | 1 | 0.734 | 1.000 |
|  | T4 - T5 | 1 | 0.940 | 0.025* |
|  |  |  |  |  |

**Supplementary table S3. Results of the contrasts between kinematic variables when the prey is on the substrate versus when the prey is suspended by tweezers in the aquatic dataset.** Symbols: ‘*’ Denotes significant interaction. Abbreviations: hd1, anterior part of the hyoid; hd2, posterior part of the hyoid; t0, initial opening of the mouth; Df, degree of freedom.

|  | Kinematic variables | DF | t.ratio | *P* |
| --- | --- | --- | --- | --- |
| Mouth movements | Maximum gape distance (MG) | 50.2 | -2.930 | 0.005* |
|  | Time to maximum gape distance (TMG) | 50.3 | -2.495 | 0.016* |
|  | Maximum gape angle (MGA) | 50.9 | -2.582 | 0.013* |
|  | Duration of the gape cycle from opening to closing of the mouth (DG) | 50.2 | -4.979 | <0.001* |
|  | Maximum speed of jaw opening (MSGO) | 50.3 | -3.917 | <0.001* |
|  | Maximum acceleration of jaw opening (MAGO) | 52.4 | -2.775 | 0.008* |
|  | Maximum speed of jaw closing (MSGC) | 50.2 | 1.268 | 0.211 |
|  | Maximum acceleration of jaw closing (MAGC) | 51.5 | 1.591 | 0.118 |
| Hd1 movements | Maximum hd1 depression (Mhd1) | 50.2 | -0.255 | 0.800 |
|  | Time to maximum hd1 depression (TMhd1) | 50.4 | -1.491 | 0.142 |
|  | Duration of hd1 cycle (Dhd1) | 50.5 | 0.918 | 0.363 |
|  | Maximum speed of hd1 depression (MSDhd1) | 52.4 | 1.012 | 0.316 |
|  | Maximum acceleration of hd1 depression (MADhd1) | 51.2 | -0.316 | 0.754 |
|  | Maximum speed of hd1 elevation (MSEhd1) | 52.4 | -0.500 | 0.619 |
|  | Maximum acceleration of hd1 elevation (MAEhd1) | 50.5 | 0.123 | 0.903 |
| Hd2 movements | Maximum hd2 depression (Mhd2) | 51.2 | -0.876 | 0.385 |
|  | Time to maximum hd2 depression (TMhd2) | 50.6 | -0.264 | 0.793 |
|  | Duration of hd2 cycle (Dhd2) | 50.3 | 0.431 | 0.669 |
|  | Maximum speed of hd2 depression (MSDhd2) | 52.4 | -0.040 | 0.968 |
|  | Maximum acceleration of hd2 depression (MADhd2) | 50.6 | 0.211 | 0.833 |
|  | Maximum speed of hd2 elevation (MSEhd2) | 50.4 | -0.609 | 0.545 |
|  | Maximum acceleration of hd2 elevation (MAEhd2) | 50.6 | -0.076 | 0.939 |
| Head movements | Maximum head angle during prey capture (MHA) | 52.4 | -3.538 | <0.001* |
|  | Time to the maximum head angle (TMHA) | 50.7 | -0.096 | 0.924 |
| Total cycle | Prey capture duration from t0 to the return of the hyoid apparatus to its initial position (PCD) | 50.2 | 0.121 | 0.905 |

**Supplementary table S4. Results of the contrasts between jaw and tongue prehension kinematics in the terrestrial dataset.** Symbols: ‘*’ Denotes significant interaction. Abbreviations: hd1, anterior part of the hyoid; hd2, posterior part of the hyoid; t0, initial opening of the mouth; Df, degree of freedom.

|  | Kinematic variables | DF | t.ratio | *P* |
| --- | --- | --- | --- | --- |
| Mouth movements | Maximum gape distance (MG) | 49.1 | -0.612 | 0.543 |
|  | Time to maximum gape distance (TMG) | 50.4 | -1.427 | 0.160 |
|  | Maximum gape angle (MGA) | 49.8 | -1.051 | 0.298 |
|  | Duration of the gape cycle from opening to closing of the mouth (DG) | 49.6 | 1.772 | 0.083 |
|  | Maximum speed of jaw opening (MSGO) | 52.6 | 0.284 | 0.777 |
|  | Maximum acceleration of jaw opening (MAGO) | 52.6 | 2.496 | 0.016* |
|  | Maximum speed of jaw closing (MSGC) | 50.7 | -1.104 | 0.275 |
|  | Maximum acceleration of jaw closing (MAGC) | 51.4 | -1.012 | 0.317 |
| Tongue movements | Maximum tongue protraction (MTgP) | 52.6 | 0.433 | 0.667 |
|  | Time to maximum tongue protraction (TMTgP) | 52.6 | -0.734 | 0.466 |
|  | Tongue movement duration (TgD) | 49.3 | 0.182 | 0.857 |
|  | Maximum speed of tongue protraction (MSTgP) | 52.6 | 0.490 | 0.626 |
|  | Maximum acceleration of tongue protraction (MAtgP) | 52.6 | 2.006 | 0.050* |
|  | Maximum speed of tongue retraction (MSTgR) | 50.9 | -0.681 | 0.499 |
|  | Maximum acceleration of tongue retraction (MAtgR) | 50.7 | 0.427 | 0.671 |
| Hd1 movements | Maximum hd1 depression (Mhd1) | 51.4 | -1.416 | 0.163 |
|  | Time to maximum hd1 depression (TMhd1) | 50.9 | -0.940 | 0.352 |
|  | Duration of hd1 cycle (Dhd1) | 52.6 | 1.175 | 0.245 |
|  | Maximum speed of hd1 depression (MSDhd1) | 51.2 | 0.689 | 0.494 |
|  | Maximum acceleration of hd1 depression (MADhd1) | 52.6 | -0.228 | 0.821 |
|  | Maximum speed of hd1 elevation (MSEhd1) | 52.6 | 0.110 | 0.913 |
|  | Maximum acceleration of hd1 elevation (MAEhd1) | 52.6 | 0.226 | 0.822 |
| Hd2 movements | Maximum hd2 depression (Mhd2) | 52.6 | 0.283 | 0.779 |
|  | Time to maximum hd2 depression (TMhd2) | 48.5 | -0.834 | 0.408 |
|  | Duration of hd2 cycle (Dhd2) | 51.1 | 1.339 | 0.187 |
|  | Maximum speed of hd2 depression (MSDhd2) | 49.1 | 0.454 | 0.652 |
|  | Maximum acceleration of hd2 depression (MADhd2) | 52.4 | 0.132 | 0.895 |
|  | Maximum speed of hd2 elevation (MSEhd2) | 52.6 | -0.053 | 0.958 |
|  | Maximum acceleration of hd2 elevation (MAEhd2) | 52.6 | 0.233 | 0.817 |
| Head movements | Maximum head angle during prey capture (MHA) | 48.6 | 0.310 | 0.758 |
|  | Time to the maximum head angle (TMHA) | 52.6 | 0.225 | 0.823 |
| Total cycle | Prey capture duration from t0 to the return of the hyoid apparatus to its initial position (PCD) | 52.6 | 1.465 | 0.149 |

**Supplementary table S5. Results of the type II ANOVA when testing for the impact of prey presentation method and medium on the first axis of the PCA.**

| Model (prey presentation method x medium) | First axis of the PCA | | | |
| --- | --- | --- | --- | --- |
|  |  | Df | *F* | *P* |
|  | prey presentation methods | 1 | 0.112 | 0.739 |
|  | Medium | 1 | 503.079 | <0.001* |
|  | Prey presentation methods x medium | 1 | 0.248 | 0.620 |
|  |  |  |  |  |

**Supplementary table S6. Results of the analysis of variance testing the impact of the medium on the main feeding kinematics contributing to the first axis of the PCA.**

| Kinematic variables | DF | F | *P* |
| --- | --- | --- | --- |
| Maximum acceleration of hd1 depression (MADhd1) | 1 | 298.300 | <0.001* |
| Time to maximum hd1 depression (TMhd1) | 1 | 296.300 | <0.001* |
| Maximum acceleration of hd2 depression (MADhd2) | 1 | 248.000 | <0.001* |
| Time to maximum hd2 depression (TMhd2) | 1 | 285.600 | <0.001* |
| Prey capture duration from t0 to the return of the hyoid apparatus to its initial position (PCD) | 1 | 356.400 | <0.001* |
| Maximum acceleration of hd1 elevation (MAEhd1) | 1 | 12.600 | <0.001* |
| Maximum acceleration of hd2 elevation (MAEhd2) | 1 | 130.500 | <0.001* |
| Time to maximum gape distance (TMG) | 1 | 979.800 | <0.001* |
| Duration of hd2 cycle (Dhd2) | 1 | 280.400 | <0.001* |
| Duration of the gape cycle (DG) | 1 | 402.000 | <0.001* |
| Maximum speed of hd1 depression (MSDhd1) | 1 | 111.700 | <0.001* |
| Duration of hd1 cycle (Dhd1) | 1 | 193.400 | <0.001* |
| Maximum acceleration of jaw closing (MAGC) | 1 | 80.480 | <0.001* |
| Maximum speed of hd2 depression (MSDhd2) | 1 | 61.700 | <0.001* |

**Supplementary figure S1. The prey presentation method significantly impacts the initial head angle of *A. mexicanum*.** Panel A displays a boxplot of initial head angle values across the two prey presentation methods. The initial head angle is higher when the prey is suspended. The maximum and minimum head angles are highlighted in red and illustrated in Panels B and C, respectively. Panel B shows the maximum initial head angle, observed in individual A3, characterized by a dorsiflexed neck posture. Panel C shows the minimum initial head angle, also observed in individual A3, where the neck is ventroflexed. Abbreviation: HA0, head angle at strike onset.


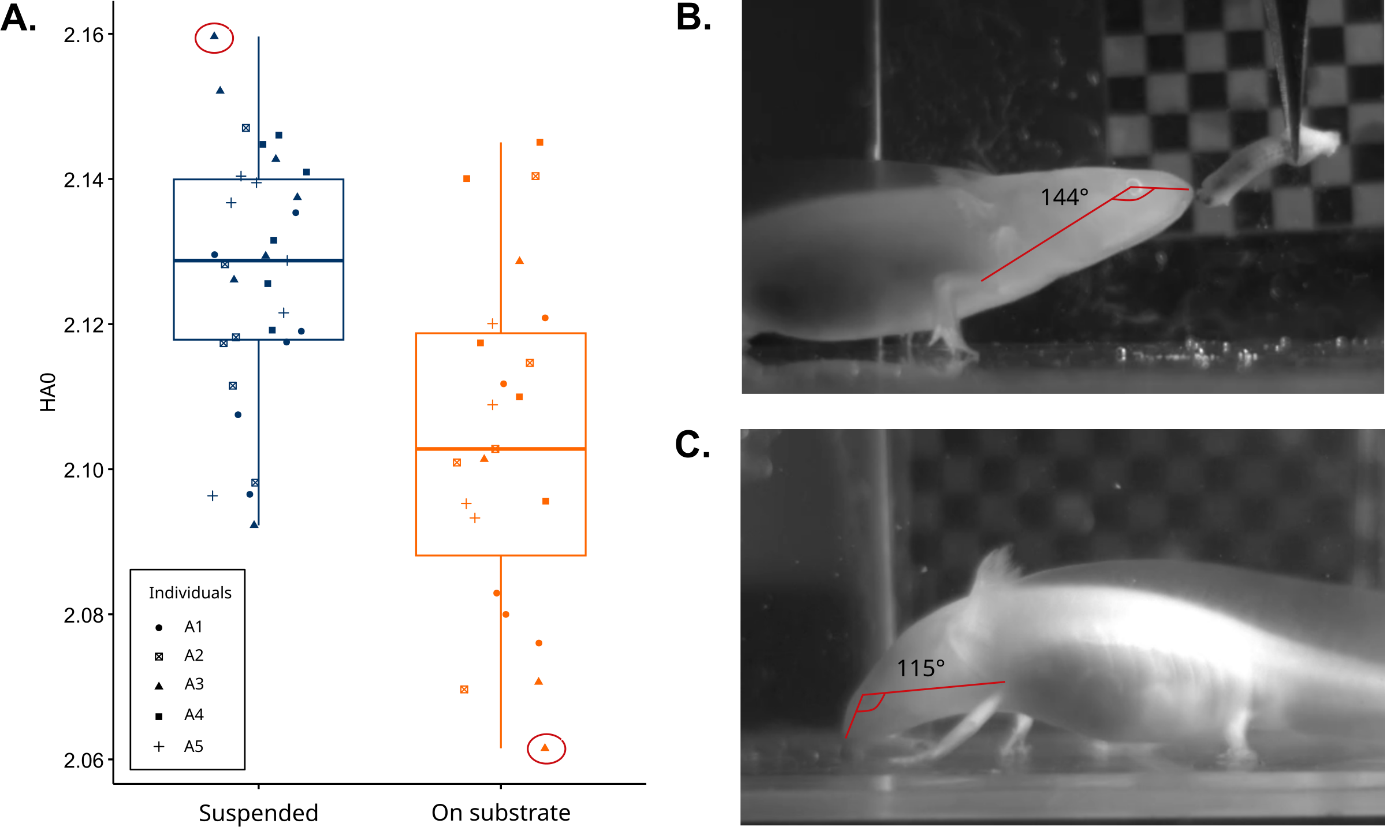


**Supplementary figure S2. Contribution of the variables to the main PC axis.** Panel A shows the contribution of variables to PC1. Panel B shows the contribution of variables to PC2. The red dashed lines indicate the expected average contribution thresholds. If all variables contributed equally to the PC axis, the expected average contribution thresholds would be 1/25 = 4%. Variables above these thresholds are considered important contributors to the PC axis. Abbreviations: MG, Maximum gape distance; TMG, Time to maximum gape distance; MGA, Maximum gape angle; DG, Duration of the gape cycle from opening to closing of the mouth; MSGO, Maximum speed of jaw opening; MAGO, Maximum acceleration of jaw opening; MSGC, Maximum speed of jaw closing; MAGC, Maximum acceleration of jaw closing; hd1, anterior part of the hyoid; hd2, posterior part of the hyoid; Mhd1-2, Maximum hd1-2 depression; TMhd1-2, Time to maximum hd1-2 depression; Dhd1-2, Duration of hd1-2 cycle; MSDhd1-2, Maximum speed of hd1-2 depression; MADhd1-2, Maximum acceleration of hd1-2 depression; MSEhd1-2, Maximum speed of hd1-2 elevation; MAEhd1-2, Maximum acceleration of hd1-2 elevation; MHA, Maximum head angle during prey capture; TMHA, Time to the maximum head angle; PCD, prey capture duration


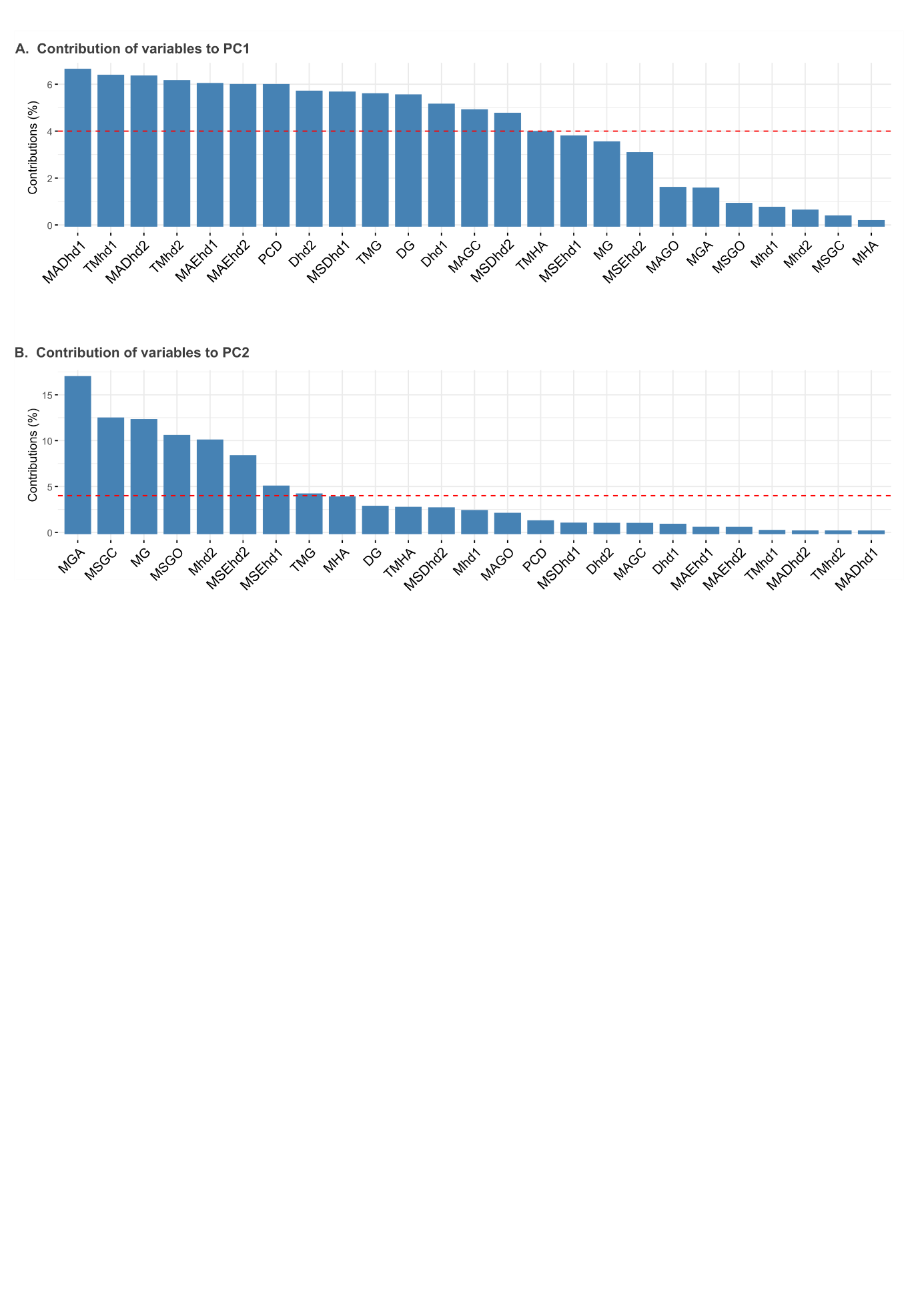


**Supplementary Movie 1. Example of a typical suction feeding sequence. (field title: SM1_gold1_free_prey_seq1_filtered_labeled; 1000fps).**

**Supplementary Movie 2. Example of a tongue prehension sequence. (field title: SM2_A_tigrinum_ind1_free_prey_seq4_filtered_labeled; 1000fps).**

**Supplementary Movie 3. Example of a jaw prehension sequence when the prey is held by tweezers. (field title: SM3_A_mavortium_ind3_tweezer_seq2_filtered_labeled; 1000fps).**

**Supplementary Movie 4. Example of a jaw prehension sequence when prey is on the substrate. (field title: SM4_A_mavortium_ind3_free_prey_seq6_filtered_labeled; 1000fps).**
